# Are patients with high-risk polycythemia vera receiving cytoreductive medications? A retrospective analysis of real-world data

**DOI:** 10.1186/s40164-018-0107-8

**Published:** 2018-07-09

**Authors:** Dilan Paranagama, Philomena Colucci, Kristin A. Evans, Machaon Bonafede, Shreekant Parasuraman

**Affiliations:** 1 Incyte Corporation, 1801 Augustine Cut-Off, Wilmington, DE 19803 USA; 2 Truven Health Analytics, an IBM Company, 75 Binney St., Cambridge, MA 02142 USA

**Keywords:** Anagrelide, Hydroxyurea, Interferons, Myeloproliferative disorders, Polycythemia vera

## Abstract

**Background:**

Patients with polycythemia vera (PV) have a higher mortality risk compared with the general population, primarily driven by cardiovascular disease, thrombotic events (TEs), and hematologic transformations. The goal of risk-adapted therapy in PV is prevention of TEs. Current treatment recommendations indicate that high-risk patients (aged ≥ 60 years and/or with history of TEs) should be managed with cytoreductive medications, phlebotomy, and low-dose aspirin. This noninterventional study was conducted to describe real-world cytoreductive medication treatment in adult patients with PV, stratified by risk, in the United States.

**Methods:**

This retrospective analysis used claims data from the Truven Health MarketScan^®^ database. Inclusion criteria were ≥ 2 nondiagnostic claims for PV ≥ 30 days apart, age ≥ 18 years, continuous enrollment during the preindex period (January 1 to December 31, 2012), and continuous enrollment or death during the postindex period (January 1, 2013, to December 31, 2014). Assessments included patient demographics, clinical characteristics, and treatment with cytoreductive medications.

**Results:**

A total of 2856 patients were identified for this analysis, including 1823 with high-risk PV and 1033 with low-risk PV. Mean (SD) age was 62.5 (13.5) years, and 65.9% of patients were male. Preindex comorbid conditions of interest were more common in high-risk than low-risk patients, including hypertension (65.0% vs 43.1%), type 2 diabetes (21.7% vs 10.1%), and congestive heart failure (6.6% vs 0.6%). Among patients who received preindex cytoreductive therapy, the most commonly used medications in high-risk (n = 666) and low-risk (n = 160) patients were hydroxyurea (94.7 and 87.5%, respectively), anagrelide (7.4 and 11.9%), and interferon (1.7 and 4.4%). Among patients who initiated cytoreductive therapy postindex, the most commonly used medications in high-risk (n = 100) and low-risk (n = 35) patients were hydroxyurea (97.0 and 91.4%, respectively), anagrelide (4.0 and 2.9%), and interferon (2.0 and 8.6%). Overall, 42.0% of high-risk and 18.9% of low-risk patients received cytoreductive medication during the preindex or postindex periods.

**Conclusions:**

Despite consistent guideline recommendations for cytoreductive therapy in patients with high-risk PV, this analysis revealed that only a minority of these patients received cytoreductive medication. A notable proportion of high-risk patients with PV would likely benefit from a revised treatment plan that aligns with current guidelines.

## Background

The Philadelphia chromosome-negative myeloproliferative neoplasms (MPNs), including polycythemia vera (PV), essential thrombocythemia, and myelofibrosis, are a group of clonal hematologic malignancies with overlapping pathology and clinical features [1]. PV is primarily characterized by erythrocytosis and constitutively active mutations in Janus kinase 2 [2]. Patients with PV commonly report chronic symptoms, including fatigue, abdominal discomfort, night sweats, concentration problems, bone pain, early satiety, and inactivity, which have a notable influence on quality of life [3]. In the recent MPN Landmark survey, the majority of patients with PV reported that their disease interfered with family or social life and that pain/discomfort associated with their disease interfered with daily activities [3].

**Fig. 1:**
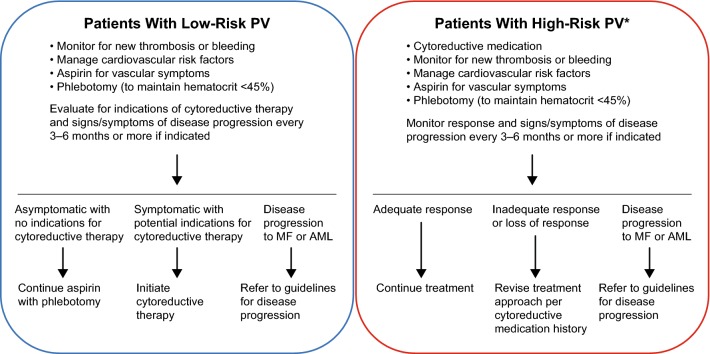
Guidelines for the treatment of low- and high-risk* patients with PV [6, 7]. *Patients with high-risk PV are ≥ 60 years of age and/or have a history of thrombotic events. *AML* acute myeloid leukemia, *MF* myelofibrosis, *PV* polycythemia vera

Patients with PV also have a higher mortality risk compared with the general population [4], primarily driven by cardiovascular disease, thrombotic events (TEs), and hematologic transformations [5]. The goal of risk-adapted therapy for patients with PV is the prevention of TEs. Treatment recommendations from the European LeukemiaNet (ELN) [6] and the National Comprehensive Cancer Network (NCCN) [7] indicate that patients with low-risk PV be managed with aspirin and phlebotomy. However, patients with high-risk PV (age ≥ 60 years and/or with a history of TEs) should be managed with cytoreductive medication in addition to phlebotomy and low-dose aspirin (Fig. 1 [6–8]). Furthermore, cytoreductive medications are recommended for patients with PV exhibiting symptomatic thrombocytosis or progressive leukocytosis, regardless of risk status [6, 7].

In the present analysis, cytoreductive medication use was evaluated in patients with PV stratified by high vs low risk to determine the extent to which real-world management aligns with guideline recommendations. This is the first study to examine real-world cytoreductive treatment practices in patients with PV relative to published treatment guidelines.

## Methods

### Patients and study design

This was a noninterventional, retrospective cohort analysis of claims from the Truven Health MarketScan^®^ database. Eligible patients lived in the United States and were ≥ 18 years of age with ≥ 2 nondiagnostic claims for PV (*International Classification of Diseases, Ninth Edition, Clinical Modification* code 238.4) ≥ 30 days apart. The index date was January 1, 2013, approximately 2 years after the ELN treatment recommendations for PV [6] were published. Patients had continuous enrollment during the preindex period (January 1 to December 31, 2012) and continuous enrollment or death during the postindex period (January 1, 2013, to December 31, 2014). Patients with claims for myelodysplastic syndrome, myelofibrosis, acute myelogenous leukemia, or secondary polycythemia were excluded.

### Assessments

Patients were stratified based on PV risk status at index, and patient demographics were assessed at the index date. Clinical characteristics, comorbidities, and concomitant medications were assessed during the preindex period. Cytoreductive medication use and phlebotomy procedures were assessed during the entire study period (i.e., preindex and postindex periods). All data were analyzed using descriptive statistics.

## Results

### Disposition and demographics

A total of 2856 patients were identified, of whom 1033 had low-risk PV and 1823 had high-risk PV. The mean age of all patients was 62.5 years and the majority were men (Table 1). Among high-risk patients, 62.8% were ≥ 60 years of age with no history of TEs, 9.1% had a history of TEs and were < 60 years of age, and 28.1% were ≥ 60 years of age and had a history of TEs.

**Table 1:** Demographics

	Low-risk PV (n = 1033)	High-risk PV (n = 1823)	All patients (N = 2856)
Mean (SD) age, years	49.7 (8.6)	69.7 (9.9)	62.5 (13.5)
Age group, n (%), years			
18–34	73 (7.1)	5 (0.3)	78 (2.7)
35–54	573 (55.5)	86 (4.7)	659 (23.1)
55–64	387 (37.5)	522 (28.6)	909 (31.8)
≥ 65	NA	1210 (66.4)	1210 (42.4)
Sex, n (%)			
Male	757 (73.3)	1125 (61.7)	1882 (65.9)
Female	276 (26.7)	698 (38.3)	974 (34.1)
Geographic region, n (%)			
South	402 (38.9)	558 (30.6)	960 (33.6)
North Central	230 (22.3)	499 (27.4)	729 (25.5)
Northeast	218 (21.1)	482 (26.4)	700 (24.5)
West	169 (16.4)	270 (14.8)	439 (15.4)
Unknown	14 (1.4)	14 (0.8)	28 (1.0)
Insurance payer, n (%)			
Commercial	1033 (100)	613 (33.6)	1646 (57.6)
Medicare	0	1210 (66.4)	1210 (42.4)
Insurance plan type, n (%)			
PPO	669 (64.8)	943 (51.7)	1612 (56.4)
Comprehensive	47 (4.5)	576 (31.6)	623 (21.8)
HMO	106 (10.3)	150 (8.2)	256 (9.0)
POS	83 (8.0)	70 (3.8)	153 (5.4)
CDHP	72 (7.0)	37 (2.0)	109 (3.8)
HDHP	32 (3.1)	13 (0.7)	45 (1.6)
EPO	6 (0.6)	18 (1.0)	24 (0.8)
POS with capitation	7 (0.7)	2 (0.1)	9 (0.3)
Unknown	11 (1.1)	14 (0.8)	25 (0.9)

Data assessed on the index date (January 1, 2013)

*CDHP* consumer-driven health plan, *EPO* exclusive provider organization, *HDHP* high-deductible health plan, *HMO* health maintenance organization, *NA* not applicable, *POS* point of service, *PPO* preferred provider organization, *PV* polycythemia vera, *SD* standard deviation

### Clinical characteristics

Out of 12 comorbid conditions, 10 were more common in high- vs low-risk patients (Table 2). Treatment with cardiovascular medication was more common in high- vs low-risk patients, whereas percentages of patients treated with corticosteroids, antidepressants, and nonsteroidal anti-inflammatory drugs were generally similar between the risk groups (Table 2).

**Table 2:** Clinical characteristics

	Low-risk PV (n = 1033)	High-risk PV (n = 1823)	All patients (N = 2856)
Comorbid condition, n (%)			
Hypertension	445 (43.1)	1185 (65.0)	1630 (57.1)
Chronic pain	187 (18.1)	473 (25.9)	660 (23.1)
Diabetes (type 2)	104 (10.1)	396 (21.7)	500 (17.5)
Osteoarthritis	102 (9.9)	366 (20.1)	468 (16.4)
Cancer (excluding leukemia and MM)	66 (6.4)	381 (20.9)	447 (15.7)
Gastroesophageal reflux disease	121 (11.7)	224 (12.3)	345 (12.1)
Anemia	64 (6.2)	168 (9.2)	232 (8.1)
Depression	81 (7.8)	123 (6.7)	204 (7.1)
Anxiety	73 (7.1)	117 (6.4)	190 (6.7)
Congestive heart failure	6 (0.6)	121 (6.6)	127 (4.4)
Non-AML leukemia	5 (0.5)	14 (0.8)	19 (0.7)
MM	1 (0.1)	8 (0.4)	9 (0.3)
Concomitant medication, n (%)			
Cardiovascular^a^	636 (61.6)	1526 (83.7)	2162 (75.7)
Corticosteroid (oral or IV)	306 (29.6)	510 (28.0)	816 (28.6)
Antidepressant	228 (22.1)	369 (20.2)	597 (20.9)
NSAID	205 (19.8)	314 (17.2)	519 (18.2)

Clinical characteristics evaluated during the preindex period (January 1 to December 31, 2012)

*AML* acute myeloid leukemia, *IV* intravenous, *MM* multiple myeloma, *NSAID* nonsteroidal anti-inflammatory drug, *PV* polycythemia vera

^a^Includes antihypertensives, statins, antidiabetic medications, anticoagulants, antiplatelet medications, and other lipid-lowering medications

### Cytoreductive medication usage

**Fig. 2:**
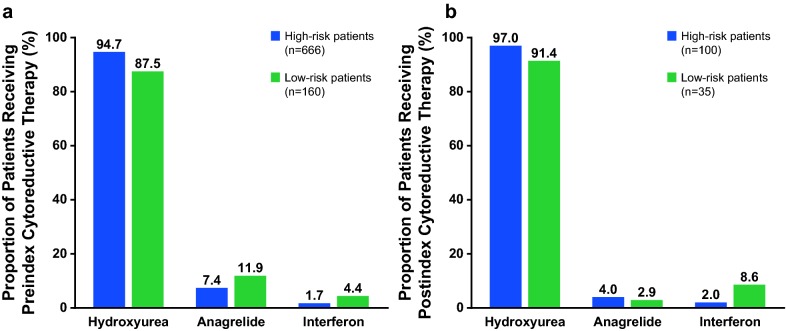
Types of **a** preindex and **b** postindex cytoreductive medications by PV risk category. **a** Data were assessed during the preindex period (January 1 to December 31, 2012). Busulfan was reported by 0.3 and 0.6% of high-risk and low-risk patients, respectively, who received preindex cytoreductive therapy. **b** Data were assessed during the postindex period (January 1, 2013, to December 31, 2014). *PV* polycythemia vera

During the preindex period, 36.5% (666/1823) of patients with high-risk PV and 15.5% (160/1033) of patients with low-risk PV received cytoreductive therapy. The most common preindex cytoreductive therapies in high- and low-risk patients were hydroxyurea (94.7 and 87.5%, respectively), anagrelide (7.4 and 11.9%), and interferon (1.7 and 4.4%; Fig. 2a).

Among patients who did not receive cytoreductive medication during the preindex period, 6.7% (135/2030) initiated a cytoreductive therapy during the postindex period; 74.1% (100/135) of whom were high risk and 25.9% (35/135) of whom were low risk. The most common postindex cytoreductive therapies in high- and low-risk patients were hydroxyurea (97.0 and 91.4%, respectively), anagrelide (4.0 and 2.9%), and interferon (2.0 and 8.6%; Fig. 2b).

**Fig. 3:**
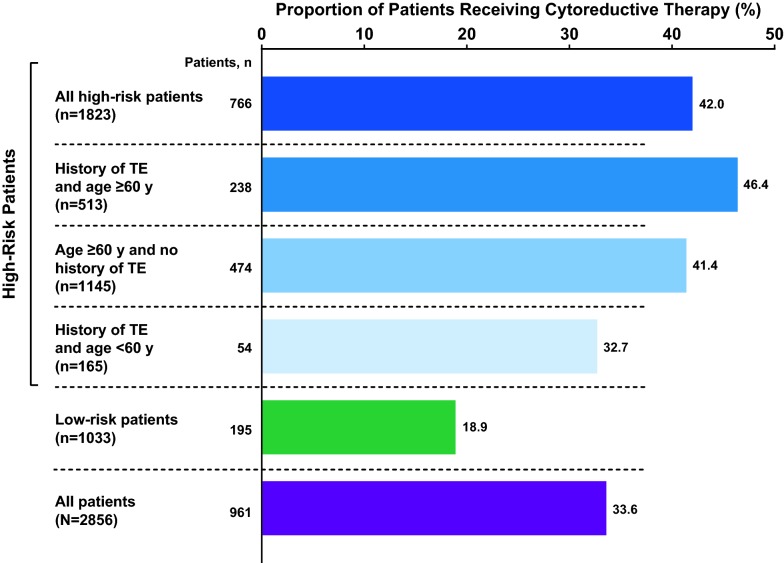
Total cytoreductive medication usage by PV risk category. Data were assessed during the preindex period (January 1 to December 31, 2012) and postindex period (January 1, 2013, to December 31, 2014) combined. *PV* polycythemia vera, *TE* thrombotic event

Overall, 42.0% (766/1823) of patients with high-risk PV and 18.9% (195/1033) of patients with low-risk PV received cytoreductive medications during either the preindex or postindex periods (Fig. 3).

### Phlebotomy procedures

A larger proportion of patients who did not receive cytoreductive therapies had phlebotomies during the preindex period compared with those who had preindex cytoreductive therapy [43.3% (880/2030) vs 37.3% (308/826), respectively]. The trend was similar in patients with high-risk PV [41.7% (483/1157) vs 36.8% (245/666)] and patients with low-risk PV [45.5% (397/873) vs 39.4% (63/160)].

## Discussion

This analysis was designed to assess real-world cytoreductive medication treatment practices in patients with PV per the ELN recommendations published in 2011 [6]. Despite current recommendations indicating that patients with high-risk PV should receive cytoreductive medication [6, 7], less than one-half of such patients (42.0%) were treated with cytoreductive medication in the present analysis. Moreover, less than one-half of high-risk patients who were both ≥ 60 years of age and had a history of TEs, and less than one-third of patients with a history of TEs who were < 60 years of age received cytoreductive medication. The nature of this retrospective database analysis precluded clinical assessments of symptoms or specific outcomes. However, patients with high-risk PV were more likely to have cardiovascular comorbid conditions (hypertension, type 2 diabetes mellitus, and congestive heart failure) and were more likely to be prescribed cardiovascular medication. The age difference between the high- and low-risk groups may have contributed to the difference in cardiovascular comorbid condition rates.

An index date of January 1, 2013, was chosen in an effort to collect data from a time period when treating physicians could be reasonably expected to follow the ELN recommendations. However, it is important to note that in July 2017, NCCN Guidelines^®^ were released pertaining to the treatment of patients with PV; these guidelines were updated in September 2017 [7]. The recommendations in the NCCN Guidelines^®^ are quite consistent with the ELN’s general recommendation that high-risk patients with PV be treated with hydroxyurea, interferons, or ruxolitinib, depending on their treatment history. Findings from this study suggest that prescribing physicians could benefit from increased awareness of consensus guidelines for the treatment of PV. Methods to improve physician education, such as increased availability of PV-focused continuing medical education materials and review articles targeting physicians, should be considered to improve guideline adherence and treatment outcomes in patients with PV.

The primary limitations associated with this study are inherent to all retrospective claims-based analyses. These include the assumption that claims were coded correctly and that patients were accurately identified as having PV (i.e., not secondary polycythemia). Furthermore, medication use was evaluated based solely on insurance claims data; the types of treating physicians who prescribed the drugs were not available in the Truven Health MarketScan database, and actual drug use during the study period was not confirmed. Finally, this study only included patients who were commercially insured or had supplemental Medicare insurance, and might not be generalizable to patients with other forms of coverage.

## Conclusions

This analysis of real-world treatment patterns indicates that most patients with high-risk PV do not receive cytoreductive medication, despite issued treatment guidelines. Given the increased mortality of patients with PV compared with age-matched subjects without PV [4], the present data suggest that a considerable proportion of patients with high-risk PV would likely benefit from revised treatment plans that align with current clinical guideline recommendations.

## Abbreviations

AML: acute myeloid leukemia
CDHP: consumer-driven health plan
ELN: European LeukemiaNet
EPO: exclusive provider organization
HDHP: high-deductible health plan
HMO: health maintenance organization
IV: intravenous
MF: myelofibrosis
MM: multiple myeloma
MPN: myeloproliferative neoplasm
NA: not applicable
NCCN: National Comprehensive Cancer Network
NSAID: nonsteroidal anti-inflammatory drug
POS: point of service
PPO: preferred provider organization
PV: polycythemia vera
TE: thrombotic event
